# Breast surgery super-specialization: Impact on advanced surgical training and research

**DOI:** 10.12669/pjms.38.8.6045

**Published:** 2022

**Authors:** Danish Ali, Sana Zeeshan, Nifasat Farooqi, Lubna Vohra

**Affiliations:** 1 Dr. Danish Ali, MBBS. Dean’s Clinical Research Fellow, Aga Khan University Hospital, Karachi, Pakistan; 2 Dr. Sana Zeeshan; MBBS, FCPS, FACS. Assistant Professor of Breast Surgery, Aga Khan University Hospital, Karachi, Pakistan; 3 Dr. Nifasat Farooqi, MBBS, FCPS. Senior Medical Officer, COVID ICU, Aga Khan University Hospital, Karachi, Pakistan; 4 Dr. Lubna Vohra, MBBS, FCPS, FACS. Assistant Professor of Breast Surgery, Aga Khan University Hospital, Karachi, Pakistan

**Keywords:** Breast Surgery, Breast Surgery Fellowship, Super-specialization, Fellowship, CPSP, FCPS

## Abstract

The earliest records of breast cancer (BC) date back to 3,000 - 2,500 B.C., ever since multiple curative options have been explored. First known wide margin excision was performed around 1^st^ Century AD and a prototype of the modern-day BC surgery, Halsted radical mastectomy, was performed in the 20^th^ Century. BC is the most common cancer in Pakistan accounting for up to 14.5% of the total cancer incidences. Accredited breast surgery fellowships were established in the UK and USA in 2002 and 2003, respectively. In Pakistan, the movement was delayed and the two-year College of Physicians and Surgeons Pakistan (CPSP) accredited breast surgery fellowship program started in 2019. The increasing annual incidence and changes in demands have led to an increased percentage of General Surgery graduates taking up specialty training. PubMed search from 1990 to 2021 showed a rising trend in the number of breast cancer publications from Pakistan; from almost no papers before 1990 to 615 between 2012 to 2021. This remarkable surge in BC-related research can be explained by the commencement of fellowship programs in breast surgery and related fields. An increase in specialist training will yield better results in the management of patients, improve clinical trials and help produce more meaningful publications from the country.

## INTRODUCTION

The Edwin Smith Surgical Papyrus (3,000-2,500 B.C.) is identified to be the earliest written and illustrated record of breast cancer (BC).^1^ Around 400 B.C. Hippocrates described the progression of BC and theorized the involvement and imbalance of ‘Humors’ (bile, phlegm, and blood). In the 1^st^ century A.D., the first known wide margin excision of breast tumor was performed and this led to the principles of modern-day surgical practice.^1^ In 1984, Professor William S. Halsted from John Hopkins Hospital in Baltimore performed the first known Halsted radical mastectomy and since then in the late 19^th^ and 20^th^ centuries with growing knowledge about hormonal involvement, improvement in imaging and surgical techniques led to less aggressive and improved outcomes.^1^,^2^

BC is now the most common cancer overall, in women, and the developed and developing parts of the world including Pakistan.^3^ In 2020, BC accounted for up to 14.5% of the total cancer incidences in Pakistan and was also the leading cause of cancer-related mortality.^4^ With one in every nine Pakistani women suffering from BC, Pakistan has the highest prevalence of BC in Asia.^5^,^6^ The age-standardized incidence rate (ASIR) of Pakistan is also one of the highest in Asia;^3^,^5^ 2.5 times higher than the neighboring countries.^6^ To tackle the increasing burden of BC in the country, a higher number of qualified and specifically trained breast surgeons is the need of the hour.

There is evidence that multidisciplinary specialist teams evaluating and managing BC result in improved outcomes.^7^ As part of their treatment, most BC patients have some type of surgery. In the last few decades, however, there has been a transition in BC management; multimodality patient-centered customized care is preferred over an initial surgery.^8^ With an ever-increasing burden of BC and a growing number of dedicated breast surgeons, it seems imperative that breast surgical oncology training should be standardized.

Breast surgical oncology training has evolved in the last two decades, but adherence to new practices is very slow. While rotating in breast surgery units, the in-training General Surgeons usually encounter limited cases and have a manpower handicap. Hence, they are not commonly exposed to a high volume of breast surgery cases.^9^

### Breast Surgery fellowships origin (Worldwide and Pakistan):

Dr. J. Harold Cheek, a General Surgeon at Baylor University Medical Center, took a keen interest in the diseases of the breast and ultimately limited his practice to the field in 1951.^10^ He observed that most Parkland surgical residents did not have adequate exposure to managing BC; such as not performing a single radical mastectomy (standard operation for BC at that time) because patients presented to the hospital at an inoperable stage.^11^ Dr. Cheek’s dream of an adequate training experience was later fulfilled by a BC patient’s generous endowment which helped establish the “Seeger Endowed Fellowship in Surgical Oncology of the Breast”. This was the first breast surgical oncology fellowship of the United States developed at the Baylor University Medical Centre in 1980.^12^ Finally, in November 2003, the first batch of in-training doctors matched in fellowship programs accredited by the American Society of Breast Surgeons (ASBS), the American Society of Breast Disease (ABSD), and the Society of Surgical Oncology (SSO).^13^ In the UK and Canada, the breast oncoplastic surgery fellowship was established in 2002.^14^

In Pakistan, this movement was delayed and the process of accreditation of breast surgical oncology fellowship began in 2019; this 2-year fellowship program is recognized by the College of Physicians and Surgeons Pakistan (CPSP).^15^ Before this, certain hospitals in the country had their own, unaccredited breast surgery fellowship. Aga Khan University Hospital (AKUH) was the first hospital in the country to start a Breast Surgery fellowship program in 1999 and since then nine fellows have graduated from the program. Currently, six out of 207 CPSP accredited institutes in the country (Aga Khan University Hospital and Liaquat National Hospital from Karachi; Shaukat Khanum Memorial Hospital & Research Centre, King Edward Medical University & Allied Hospital, and Ittefaq Hospital from Lahore, and Combined Military Hospital from Rawalpindi) are fully licensed to train graduated General Surgeons in managing surgical diseases of the breast.^16^

In The International Surgical Week 2007 conference held in Montreal, well-known breast surgeons from across the globe representing both the developing and developed world presented on the development of oncoplastic breast surgery. Data from Australia showed that 1200 active General Surgeons, offered breast services, and 20% exclusively practiced breast surgery. Croatia’s model, considered to be the most successful, utilized a General Surgeon knowledgeable about principles of both oncologic and plastic surgery who acted as the team leader. In India, a vast majority of patients were treated by General Surgeons, and tumor ablation via modified radical mastectomy was the main goal. Owing to a shortage of plastic surgeons oncoplastic procedures were offered to only a handful of patients.^9^ Currently, mastectomy is still considered the mainstay of treatment but in select cases, surgeons have keenly acknowledged the use of BCS.^17^ With growing demand and popularity in breast surgery; there has been a rising trend in research on the topic and breast oncoplastic training has now been incorporated in specialist surgical oncology training in India.^17^

With changing expectations and roles of surgeons, it is imperative that in-training breast surgeons are not only technically sound but also possess the leadership qualities to head a multidisciplinary team, be a patient’s first contact, guide the patient’s management according to the latest guidelines and be updated with the latest developments in the academic and clinical aspects of their field. Such expectations can be fulfilled by the formation of an optimal training program that allows breast and plastic surgeons to help their patients safely and achieve good outcomes. This can be done by choosing an apprenticeship method of surgical training. This method, which is now considered to be the gold standard incorporates guided surgeries, simulations, and cadaver labs.^18^ Furthermore, surgeons all over the world are now expected to continuously improve their knowledge and adapt to the latest treatment options.^19^

### Do we need Breast Surgery as a specialization?

In the coming two decades if the trends of cancer incidence and rates of oncological procedures remain unchanged, the number of oncological procedures will increase by almost 24% to 51%.^20^ General surgeons are well trained in different oncological procedures; however, it is counter-intuitive to expect that they might be able to handle an increased number of site-specific oncological cases. This is because they will experience a similar increase in their practice.^21^

The incidence of BC in Pakistan is the highest of any Asian country; the common presentations of BC occur at a more advanced stage and younger age.^22^,^23^ Along with high incidence, there is also a delay in patient diagnosis which is assumed to be due to an inadequately trained number of specialists for the disease.^24^ Hence, the burden of high patient load shifts to surgeons who are not specialists for managing breast diseases: general surgeons and gynecologists. This leads to suboptimal treatment and hence, becomes an avoidable cause of psychological and physical morbidity for the patients.^24^ Due to a shortage of specialist doctors in almost all fields, breast surgeons commonly play an all-important in the management of BC patients by leading the multi-modal treatment.^24^ Therefore, Pakistan requires a higher number of trained breast surgeons to adequately manage the ever-growing burden of BC.

The general population is now more aware of subspecialized fields and General Surgeons have responded to the increase in demand by seeking specialty training in record numbers. A similar trend is also seen in our part of the world. In the USA, from 2002-2013 almost three-quarters of General Surgery graduates undertook subspecialty training, with 6.8% specializing in Surgical Oncology and 4.7% in Breast Surgery. This is a major increase from 1983 to 1990 when only half of General Surgery graduates pursued specialist training.^25^,^26^

The general population also perceives that a specialist surgeon can deliver better services than a General Surgeon. Centers with a high volume of cases have a lesser mortality rate for complex oncological surgeries, this has been corroborated by many studies and also observed in BC management as reported by Roohan et. Al.^27^ It has been observed that surgeons specializing in breast surgery had a 16%-33% lower 5-year risk of mortality compared to surgeons who did not specifically specialize in breast surgery.^28^ Skinner et al.^29^ concluded that this difference existed because surgical oncologists performed breast-conserving procedures significantly more often.

Currently, the competency gap for the entirety of surgical procedures is not well ascertained between subspecialized surgeons and their non-specialized counterparts. General surgeons learn and perform a wide variety of surgical procedures and their range of procedures varies with the size of the community.^30^ They are well known to do the majority of colorectal procedures;^31^ however, the competency gap was notable only while performing advanced colorectal procedures.^32^ A reduction in postoperative complications was observed for advanced hepatobiliary procedures^33^ and a reduction in morbidity and mortality was seen amongst pediatric-urology patients when a specialist surgeon performed the procedures.^34^ However, no such significant difference was observed in laparoscopic appendectomies^35^ apart from the fact that the conversion to an open procedure was more commonly observed in non-specialized surgeons.^35^,^36^ A systematic review performed by Johnston et al. concluded that fellowship training does have a positive impact on patient outcomes.^36^ Hence, from the available data, it is safe to conclude that General Surgeons are well-equipped with performing a wide array of surgical procedures but when special circumstances demand advanced surgical care, fellowship-trained surgeons can help improve patient outcomes.

With an already high ASIR, a recent increase in BC cases in younger patients, and common presentation of BC at an advanced stage of the disease, the importance of early detection and management of cancer has become of grave importance. The disparity in breast surgical oncological care is evident by the fact that in 2020 a total of 25,928 new BC cases were diagnosed in Pakistan, while there are only 20 cancer hospitals to deal with it.^37^ With a scarcity of trained breast surgeons and an enormous demand to be met, most cases of BCs in rural and semi-urban areas are dealt with by either General Surgeons or Gynecologists. Increasing Breast Cancer surgery centers and the number of accredited fellowship programs will help improve surgical care and outcomes in BC patients.

### Impact of breast surgical training on research:

**Fig.1:**
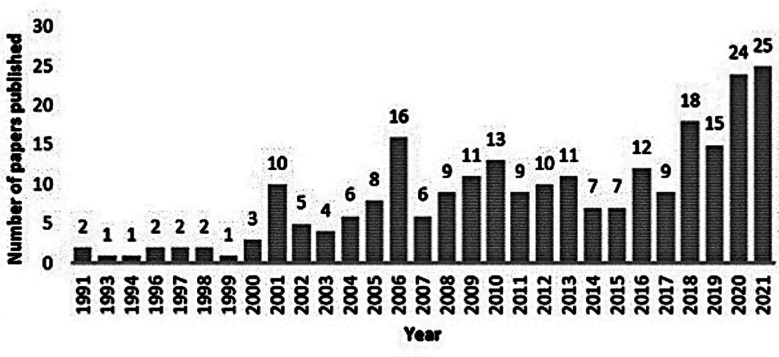
Graph showing number of yearly publications on breast disease from Aga Khan University Hospital from 1991-2021 reflecting gradual increase from the initiation of specialty training in 1999.

With the acceptance and evolution of breast surgical training as a separate specialty, a positive influence has been observed on research from Pakistan. There has been a rising trend of published research articles from all over Pakistan, especially from AKUH since 1990. Upon PubMed search, only one article related to breast diseases was published from Pakistan before 1990, from 1990 to 2000, a total of 18 papers were published in peer-reviewed journals on various aspects of breast diseases, and the numbers grew tremendously since then. There were a total of 152 papers published from 2001 to 2011 and 615 from 2012 to 2021 with the most papers being published in 2020 followed by 2019. In the last 30 years, a total of 249 articles related to various aspects of breast diseases were published from AKUH, with more than half of the articles being published in the last decade (2011-2021). Since the unaccredited breast surgery fellowship was initiated at AKUH in 1999, the number of publications has grown tremendously since then (Fig.1). This remarkable increase of interest in BC research in Pakistan can be explained by the commencement of fellowship programs in Breast Surgery, Radiation Oncology, and Diagnostic Radiology programs albeit unaccredited until recent times.

There are few societies across the globe working for improvements and innovations in breast surgery. The American Society of Breast Surgeons (ASBrS) is one such example, as a part of its mission, the ASBrS has been edifying the science of breast surgery for specialist surgeons via the promotion of research, training, development of advanced surgical techniques, and forming a convention for exchange of new ideas.^38^ The other Major society is the ESSO – EUSOMA survey for the development of breast training across Europe.^28^

## CONCLUSION

Practicing multidisciplinary care, management of BC at a high-volume center, and by a breast specialist has improved patient outcomes. This leads to the subsequent belief that specialized training at high-frequency centers will result in improved technical and surgical prowess amongst breast surgery trainees. This in turn will promote research, leading to better trials and meaningful publications from the country resulting in improved knowledge and approach.

### Authors’ Contribution:

**SZ:** Conceived and designed the project. **DA & SZ:** Are responsible for writing the manuscript and the integrity of the research. **NF, SZ & LV:** Edited the manuscript. **SZ & LV:** Reviewed and gave the final approval of the manuscript.
